# Transcriptome profiling reveals the response process of tomato carrying Cf*-19* and *Cladosporium fulvum* interaction

**DOI:** 10.1186/s12870-019-2150-y

**Published:** 2019-12-19

**Authors:** Tingting Zhao, Wenhong Liu, Zhentong Zhao, Huanhuan Yang, Yufang Bao, Dongye Zhang, Ziyu Wang, Jingbin Jiang, Ying Xu, He Zhang, Jingfu Li, Qingshan Chen, Xiangyang Xu

**Affiliations:** 1 College of Horticulture and Landscape Architecture, Northeast Agricultural University, Harbin, 150030 China; 2 College of Agronomy, Northeast Agricultural University, Harbin, China; 3 Key Laboratory of Biology and Genetic Improvement of Horticultural Crops (Northeast Region), Ministry of Agriculture and Rural Affairs, Harbin, China

**Keywords:** Cf*-19* gene, *Solanum lycopersicum*, *Cladosporium fulvum*, Tomato leaf mould, RNA-seq, Resistance response

## Abstract

**Background:**

During tomato cultivation, tomato leaf mould is a common disease caused by *Cladosporium fulvum* (*C. fulvum*). By encoding Cf proteins, which can recognize corresponding AVR proteins produced by *C. fulvum*, Cf genes provide resistance to *C. fulvum*, and the resistance response patterns mediated by different Cf genes are not identical. Plants carrying the Cf*-19* gene show effective resistance to *C. fulvum* in the field and can be used as new resistant materials in breeding. In this study, to identify key regulatory genes related to resistance and to understand the resistance response process in tomato plants carrying Cf*-19*, RNA sequencing (RNA-seq) was used to analyse the differences between the response of resistant plants (CGN18423, carrying the Cf*-19* gene) and susceptible plants (Moneymaker (MM), carrying the Cf*-0* gene) at 0, 7 and 20 days after inoculation (dai).

**Results:**

A total of 418 differentially expressed genes (DEGs) were identified specifically in the CGN18423 response process. Gene Ontology (GO) analysis revealed that GO terms including “plasma membrane (GO_Component)”, “histidine decarboxylase activity (GO_Function)”, and “carboxylic acid metabolic process (GO_Process)”, as well as other 10 GO terms, were significantly enriched. The “plant hormone signal transduction” pathway, which was unique to CGN18423 in the 0–7 dai comparison, was identified. Moreover, ten key regulatory points were screened from the “plant hormone signal transduction” pathway and the “plant pathogen interaction” pathway. Hormone content measurements revealed that the salicylic acid (SA) contents increased and peaked at 7 dai, after which the contents deceased and reached minimum values in both CGN18423 and MM plants at 20 dai. The jasmonic acid (JA) content increased to a very high level at 7 dai but then decreased to nearly the initial level at 20 dai in CGN18423, while it continued to increase slightly during the whole process from 0 to 20 dai in MM.

**Conclusions:**

The initial responses are very different between the resistant and susceptible plants. The “plant hormone signal transduction” pathway is important for the formation of Cf*-19*-mediated immunity. In addition, both JA and SA play roles in regulating the Cf*-19-*dependent resistance response.

## Background

Tomato (*Solanum lycopersicum*; *S. lycopersicum*) leaf mould is an economically important disease that causes considerable yield losses in tomato cultivation worldwide. This disease is caused by the biotrophic fungus *Cladosporium fulvum* (*C. fulvum*) [1], which exists as many different physical races and differentiates rapidly. *C. fulvum* resistance genes (Cf genes), which provide resistance to *C. fulvum*, were identified in wild species and subsequently bred into cultivated tomato. The use of Cf genes in tomato breeding represents an efficient method for controlling leaf mould disease in tomato cultivation [2, 3].

Plants have evolved two kinds of innate immune mechanisms: PAMP-triggered immunity (PTI) and effector-triggered immunity (ETI) [4, 5]. PTI is a resistance reaction activated by the pattern recognition receptors (PRR) to identify conserved pathogen-associated molecular patterns (PAMPs). ETI was shown to depend on plant resistance proteins (R proteins) to identify pathogen-secreted proteins directly or indirectly and to activate a strong resistance reaction inhibiting pathogen infection [6].

Tomato and *C. fulvum* are model pathosystems for the study of ETI [1, 7, 8]. The distinct recognition mechanism between Cfs and Avrs, including Cf-2/Avr2 [9] and Cf-9/Avr9 [10], has already been reported. Many studies have focused on Cf/Avr-dependent defence responses. Both Avr4/Cf-4 and Avr9/Cf-9 signalling elicit responses by the same spectrum of genes, while the Avr4/Cf-4-dependent hypersensitive response (HR) is more severe than the Avr9/Cf-9-dependent HR, indicating that the distinction between the Avr4/Cf-4- and Avr9/Cf-9-dependent HR most likely results from events upstream of the defence responses [11, 12]. Studies have shown that, in the Cf*-12*-mediated resistance response process, differentially expressed genes (DEGs) were significantly enriched in defence-signalling pathways such as the calcium-dependent protein kinase (CDPK) pathway and the jasmonic acid (JA) signalling pathway [13], and in the Cf*-10*-mediated resistance response process, the majority of DEGs were associated with defence-signalling pathways, including those involving oxidation-reduction processes, oxidoreductase activity and plant hormone signal transduction [14]. Additionally, many transcription factor (TF) genes were also identified in Cf*-12* and Cf*-10* resistance-related DEGs, indicating that TFs play an important role in the *C. fulvum* defence response [2].

Cf genes encode receptor-like proteins (RLPs), which are type I transmembrane glycoproteins that contain extracellular leucine-rich repeats (eLRRs), a transmembrane region, and a short cytoplasmic domain that exhibits no similarity to known signalling domains [1]. By recognizing Avr peptides that are secreted into the leaf apoplast during infection, Cf gene products provide resistance to specific races of *C. fulvum* [1]. To date, there have been at least 25 physical races of *C. fulvum* identified, and 10 effectors (Avr2, Avr4, Avr4E, Avr9, Ecp1, Ecp2, Ecp4, Ecp5, Ecp6 and Ecp7) have been cloned [15–23]. Twenty four Cf genes have been mapped onto 12 chromosomes, and 6 Cf genes (Cf*-2* (Hcr2-2B, Hcr2-2C), Cf*-5*, Cf*-4* (Hcr9-4D), Cf*-4E* (Hcr9-9B), Cf*-9* (Hcr9-9C), *9 DC* (Hcr9-M205) [18–22, 24] and 4 Cf*-Ecp* genes (Cf*-Ecp1*, Cf*-Ecp2*, Cf*-Ecp3* and Cf*-Ecp5*) have been cloned [2, 25, 26].

The Cf-*19* gene is a very efficient resistance gene, with no infection reports to date. This gene has been mapped to the short arm of chromosome 1, and a candidate gene, Solyc01g006550.2.1 (an allele of Cf*-0*), was screened within the Cf*-4/9* locus as a novel homologue of the *Cladosporium* resistance gene Cf*-9* (*Hcr9*) [27]. Our previous study also showed that Cf*-19* induced a marked HR in tomato plants inoculated with *C. fulvum* physiological race 1.2.4.5 [28]. In that study, we utilized the cDNA-amplified fragment length polymorphism (cDNA-AFLP) technique combined with bulk segregant analysis (BSA) to isolate tomato genes related to the Cf*-19*-mediated resistance response. Twenty-six differentially expressed transcript-derived fragments (TDFs) that were upregulated in the Cf*-19*-mediated HR process were isolated. The expression patterns of most of the genes detected by the transcript profiling of the Cf*-19*-mediated defence response were similar between the Cf*-4-* and Cf*-9*-mediated defence responses. *SAMDC* (TDF35) and *Eli3* (TDF49) were first detected in the Cf*/Avr* interaction [28]. Because of the limitation of cDNA-AFLP technology, we did not obtain enough information from the study. With the development of second-generation sequencing technology, RNA sequencing (RNA-seq) has become an efficient way for the analysis of host–pathogen interactions. The successful application of RNA-seq in plant host–pathogen interaction analyses, especially in tomato (carrying Cf*-12* or Cf*-10*)-*C. fulvum* [13, 14] and tomato-*Stemphylium lycopersici* [29] interaction analyses, allows a feasible way to study the Cf*-19*-mediated resistance response process. In the present study, RNA-seq technology will be used to analyse the Cf*-19*-mediated resistance response process. The results will help us further understand tomato defence mechanisms and will provide a basis for discovering new ways of controlling tomato leaf mould disease.

## Results

### Artificial inoculation

**Fig. 1:**
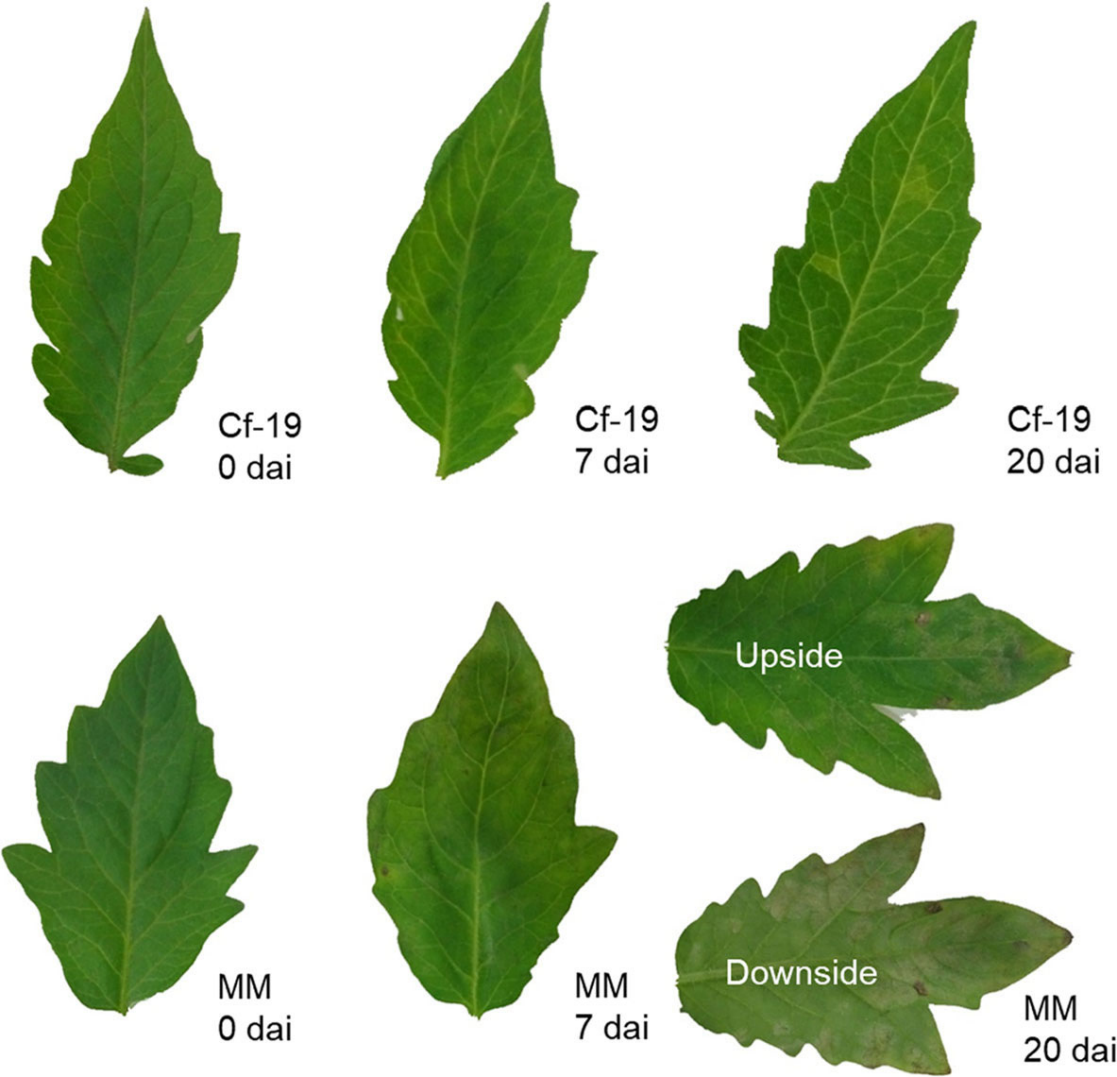
*C. fulvum* infection symptoms on the leaves of CGN18423 and MM plants. No notable signs were observed on Cf-19 leaves at 7 dai, while significant chlorotic spots were observed on Cf-19 leaves at 20 dai. Slight chlorosis and shrinkage were observed for MM leaves at 7 dai; abundant mould grew on both sides of MM leaves at 20 dai. Cf-19: CGN18423 plants; MM: Moneymaker plants. dai: days after inoculation

As shown in Fig. 1, at 7 days after inoculation (dai), no notable signs were observed on the leaves of CGN18423 (Cf*-19*), while the leaves of the susceptible line Moneymaker (MM) became chlorotic and slightly wilted. At 20 dai, some significant chlorotic spots, which are considered HR signs, were observed on the leaves of CGN18423 plants, and abundant mould grew on both sides of MM leaves. These classic symptoms indicated that our artificial inoculation was successful and that the plant materials collected at different time points could be used for subsequent analyses.

### Illumina sequencing, read mapping and identified transcripts

In this project, 18 samples were sequenced using an Illumina HiSeq platform, with an average of 4.40 Gb generated. More than 97% of reads were Q > 20, and 92% of these clean reads were > Q30 (Additional file 1: Table S1). The data with a quality score > Q30 were used for subsequent analyses. After filtering, an average of 29.32 million clean reads was generated, and at least 85% of these reads were mapped to the tomato reference genome (ftp://ftp.solgenomics.net/tomato_genome/assembly/build_3.00/); of these, more than 83% of the clean reads were uniquely mapped reads. After transcript reconstruction, a total of 35,136 novel transcripts, including 17,690 unknown splicing events for known genes, 1123 novel coding transcripts without any known features, and 16,323 long noncoding RNAs were obtained.

### DEGs in response to *C. fulvum* inoculation

**Fig. 2:**
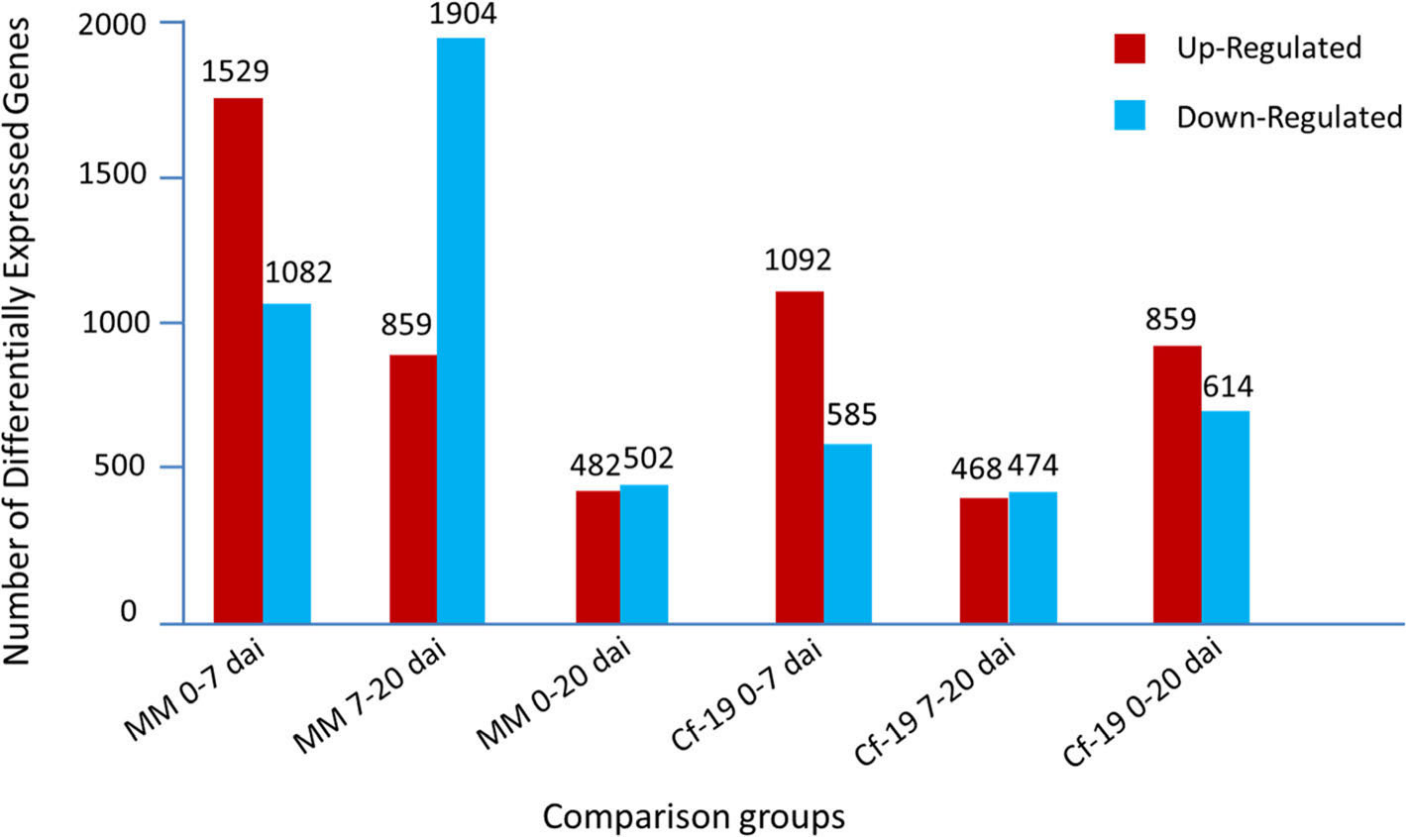
Statistics of DEGs that exhibit different expression patterns. 0–7 dai: comparison between 0 and 7 dai; 7–20 dai: comparison between 7 and 20 dai; 0–20 dai: comparison between 0 and 20 dai; dai: days after inoculation

DEGs between samples (Fig. 2) were detected based on the gene expression results. During the first stage from 0 to 7 dai, more upregulated DEGs than downregulated DEGs were detected in both MM and CGN18423 plants. During the second stage from 7 to 20 dai, fewer upregulated DEGs than downregulated DEGs were detected in both MM and CGN18423 plants. During the whole plant-pathogen interaction process, downregulated DEGs were much more prevalent than upregulated DEGs in MM, while upregulated DEGs were much more prevalent than downregulated DEGs in CGN18423 plants.

### Verification of DEG expression patterns

**Fig. 3:**
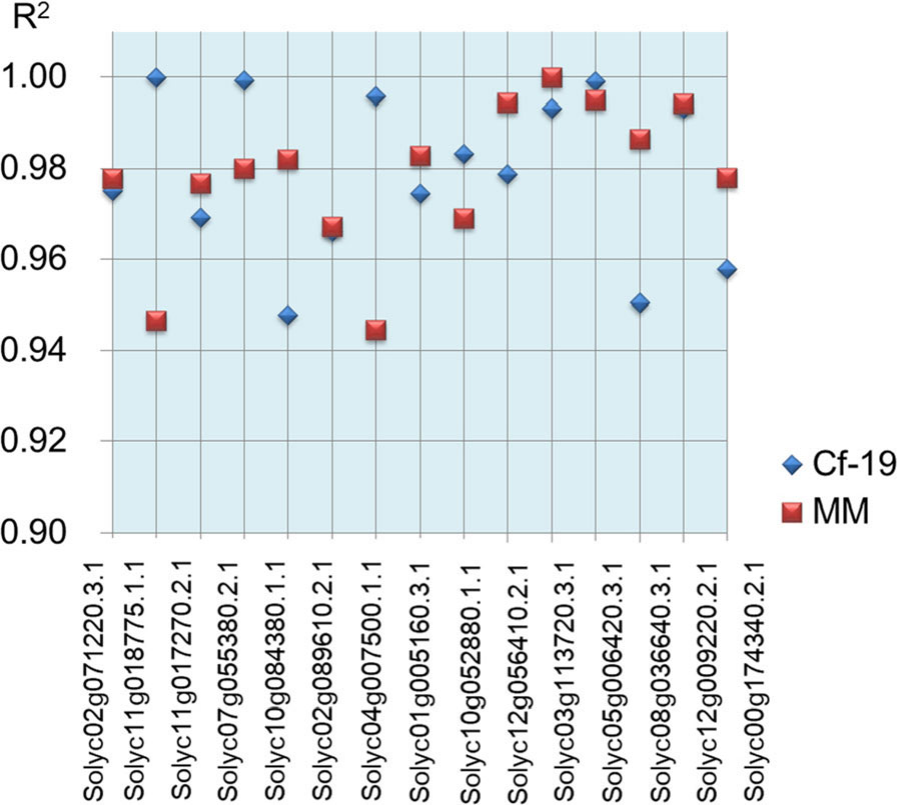
Correlation coefficients between the RNA-seq and qRT-PCR results. The expression patterns for both Cf-19 (0, 7 and 20 dai) and MM (0, 7 and 20 dai) were analysed for each gene. The results obtained from both methods (RNA-seq and qRT-PCR analysis) were used to calculate correlation coefficients (R^2^ values). Each point in the figure represents an “R^2^” value. MM: Moneymaker; Cf-19: CGN18423

To verify the accuracy of the DEG expression patterns in RNA-seq data, quantitative real-time PCR (qRT-PCR) was used to analyse the expression patterns of 15 genes that were selected randomly from the DEGs. The plant samples used for RNA-seq, which included leaves collected from CGN18423 and MM plants at 0, 7 and 20 dai, were used for this analysis. Expression patterns for both CGN18423 (0, 7 and 20 dai) and MM (0, 7 and 20 dai) were analysed for each gene. Correlation coefficients between the RNA-seq and qRT-PCR results were calculated, and the results showed that the pairwise correlation coefficients (R^2^ values) were between 0.94 and 1.0 (Fig. 3). These results reflect the high quality of our RNA-seq data, which could be used in subsequent analyses.

### Gene ontology (GO) enrichment and Kyoto encyclopedia of genes and genomes (KEGG) pathway analyses

**Fig. 4:**
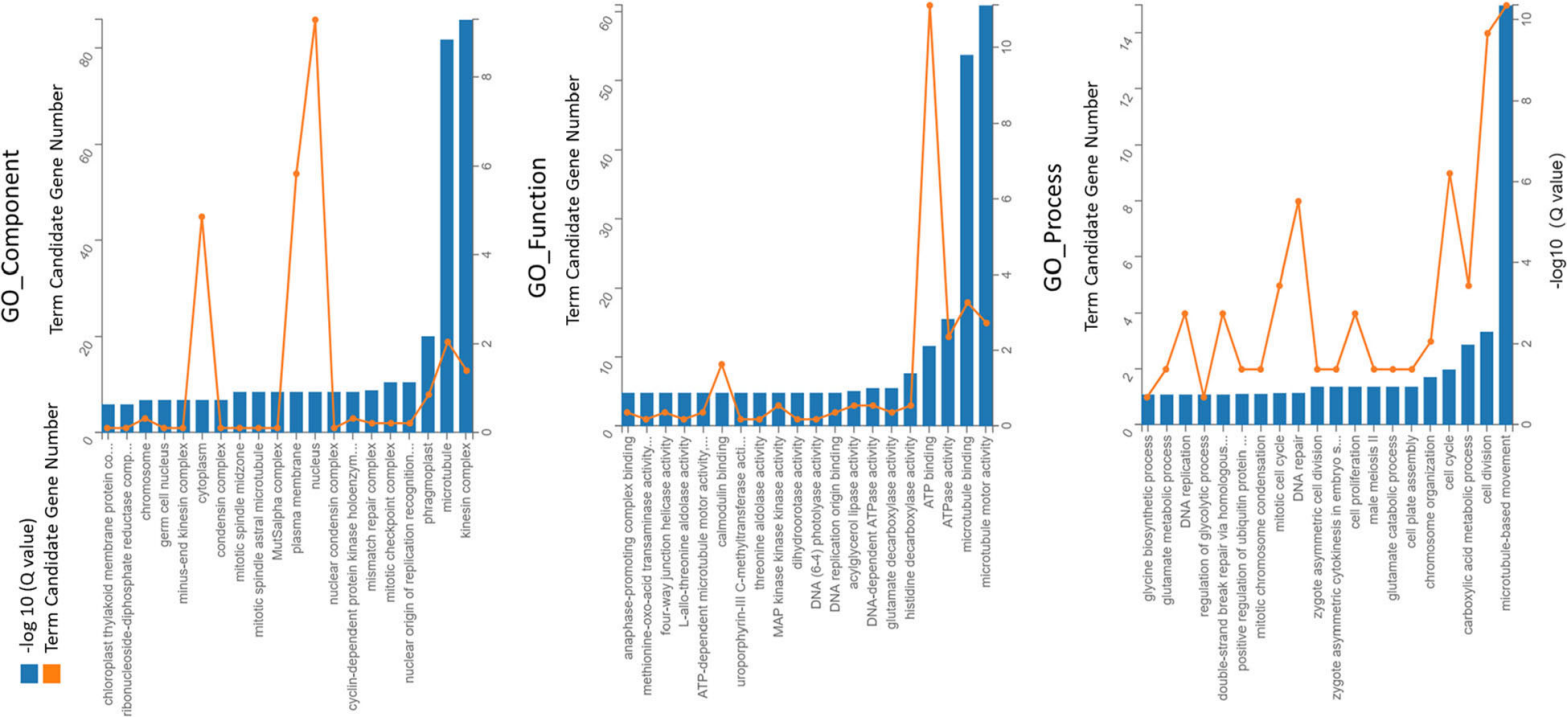
GO enrichment of 418 differentially expressed genes (DEGs) identified specifically in the CGN18423 response process

The GO classification results are shown in Additional file 3: Figure S1. DEGs at different stages of different plants were enriched in three ontology categories: biological process, cellular component and molecular function. Compared with CGN18423, MM presented more genes associated with almost all GO terms, except for the GO term “cell killing” in the 7–20 dai comparison. The number of genes associated with most GO terms, which increased in the second stage of plant-pathogen interactions in MM plants, decreased in CGN18423 plants. A total of 418 DEGs were identified specifically in the CGN18423 response process. GO analysis revealed that GO terms including “kinesin complex (GO_Component)”, “microtubule motor activity (GO_Function)”, and “microtubule-based movement (GO_Process)”, as well as 10 others (Q value <0.05), were significantly enriched (Fig. 4).

**Fig. 5:**
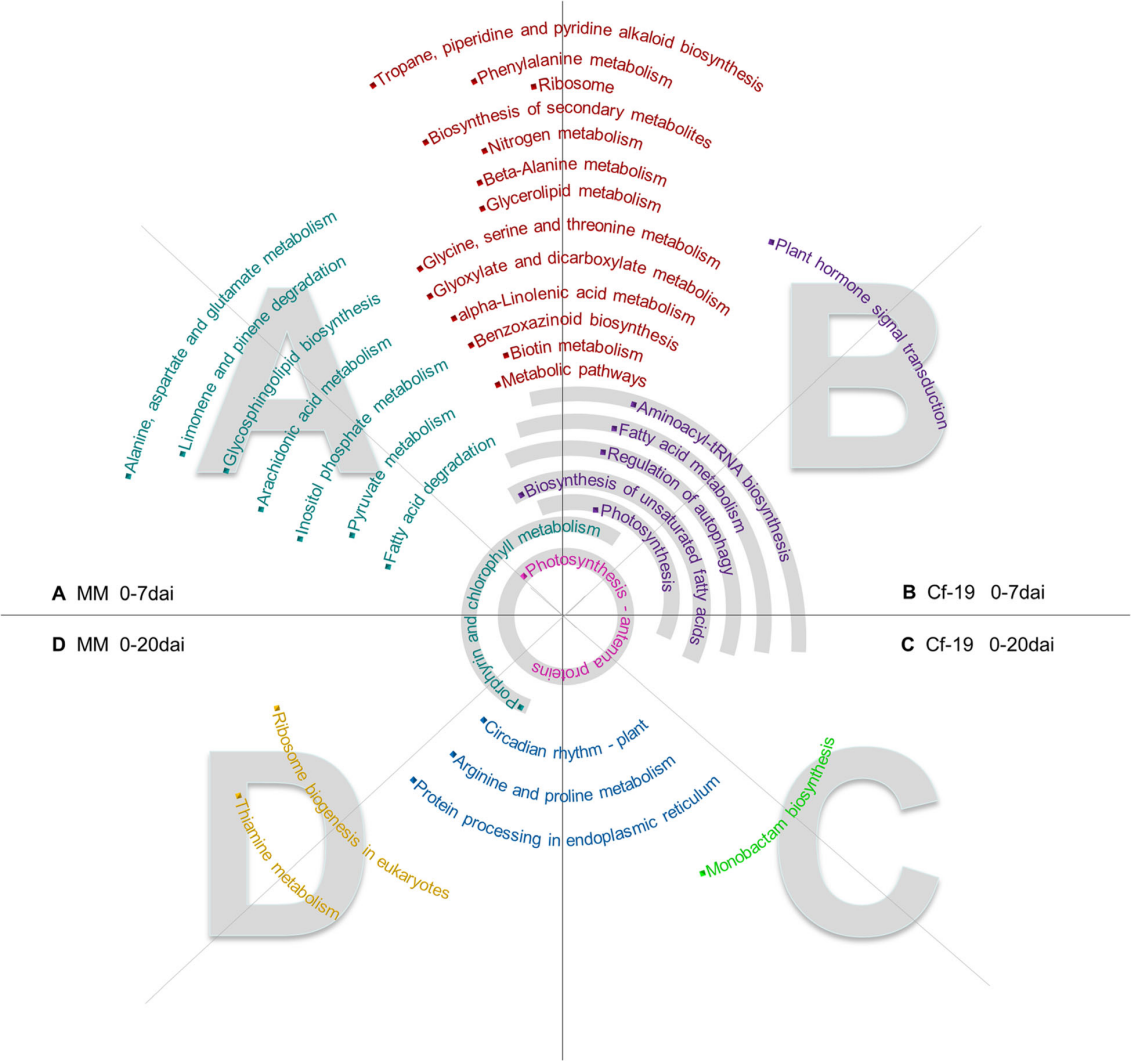
Comparison and analysis of the main pathways within the different samples. There are four parts of the figure: A, B, C and D. If pathway names cover different parts, that pathway can be found in the samples represented by the overlapping parts

Pathways with *P*-values < 0.05 were screened and used for comparative analyses. As shown in Fig. 5, more pathways were detected in the 0–7 dai comparison than in the 0–20 dai comparison in both MM and CGN18423 plants. Twenty pathways were common between the MM and CGN18423 plants in the 0–7 dai comparison, and 4 common pathways were detected between MM and CGN18423 plants in the 0–20 dai comparison. Seven and two pathways were unique to MM in the 0–7 dai and 0–20 dai comparisons, respectively. The samples of CGN18423 at both of those stages had only one unique pathway. The “plant hormone signal transduction” pathway, which plays an important role in plant resistance to pathogens, is unique to CGN18423 in the 0–7 dai comparison.

### Identification of key regulatory points from important pathways

“Plant hormone signal transduction” was the only pathway that was unique to CGN18423 in the 0–7 dai comparison; moreover, because “plant pathogen interaction” is an important pathway in all plant-pathogen interaction studies, we focused on these two pathways in this section. Pathway regulatory points containing genes that exhibit upregulated expression patterns in CGN18423 in the 0–7 and 0–20 dai comparisons (vertical comparisons) or that exhibit upregulated expression patterns in CGN18423 plants compared to MM plants at the same interaction stages (lateral comparisons) were identified as important regulatory points. The details of the comparison process and information are indicated on the pathway maps from the KEGG analysis (map04075, plant hormone signal transduction; map04626, plant-pathogen interaction). As shown in Additional file 4: Figure S2, “SAUR” in the “tryptophan metabolism” pathway, “A-ARR” in the “zeatin biosynthesis” pathway, “BRI-1” in the “brassinosteroid biosynthesis” pathway and “JAZ” in the “α-linolenic acid metabolism” pathway were identified from the “plant hormone signal transduction” pathway based on the above-mentioned resistance-related expression patterns. In the “plant pathogen interaction” pathway (Additional file 5: Figure S3), “CaMCML”, “WRKY25/22”, “WRKY28/33”, “CERK1”, “RRS1-R” and “WRKY1/2” were identified.

### Analysis of gene expression patterns at key regulatory points

**Fig. 6:**
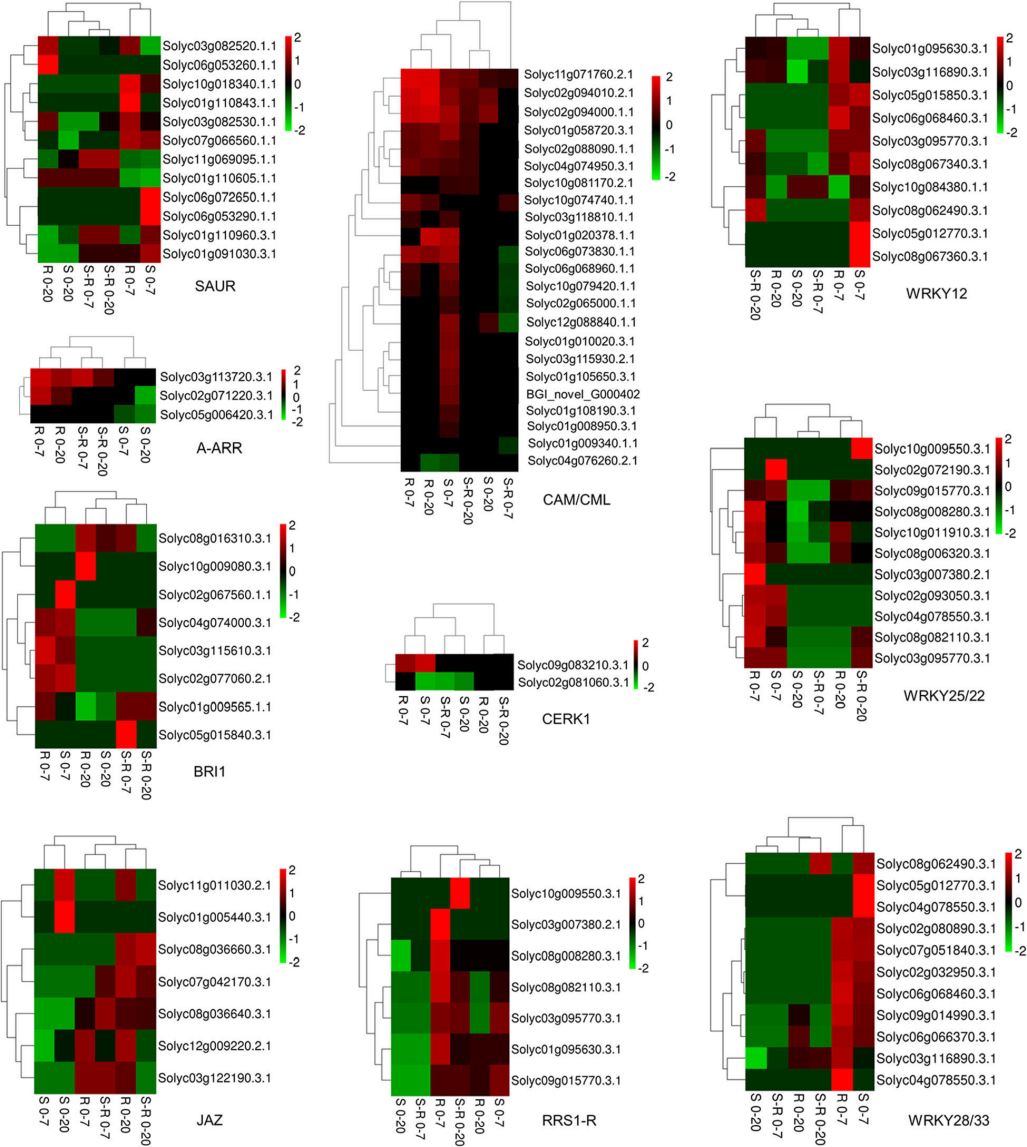
Expression pattern of genes at key regulatory points

Further analysis of the gene expression patterns at the key regulatory points was carried out. The detailed expression information is shown in Fig. 6 as heat maps. Genes at different regulatory points exhibited different expression patterns among 6 comparisons. Most genes exhibited significantly upregulated expression patterns in the “R 0–7”, “R 0–20”, “S-R 0–7” and “S-R 0–20” comparisons, especially in the “R 0–7” comparison. Of all the regulatory points in the “plant hormone signal transduction pathway”, genes associated with “A-ARR” and “JAZ” exhibited more uniform gene expression pattern cluster results, which means that the response patterns of the genes at these two points were similar (upregulated or downregulated) and that the genes may have similar functions. Of all the regulatory points in the “plant pathogen interaction pathway”, “CaM/CML” is the largest point, containing 23 DEGs. The expression patterns of most genes at this point were upregulated in the 0–7 dai comparison in both the susceptible and resistant plants and in the 0–20 dai comparison in the resistant plants. Three WRKY-related regulatory points, which contain 24 genes in total, were identified in the “plant pathogen interaction pathway”. Most WRKY genes exhibited significantly upregulated expression patterns in the 0–7 dai comparisons, while the upregulation of the same genes was more significant in the resistant plants than in the susceptible plants.

### Hormone measurements

**Fig. 7:**
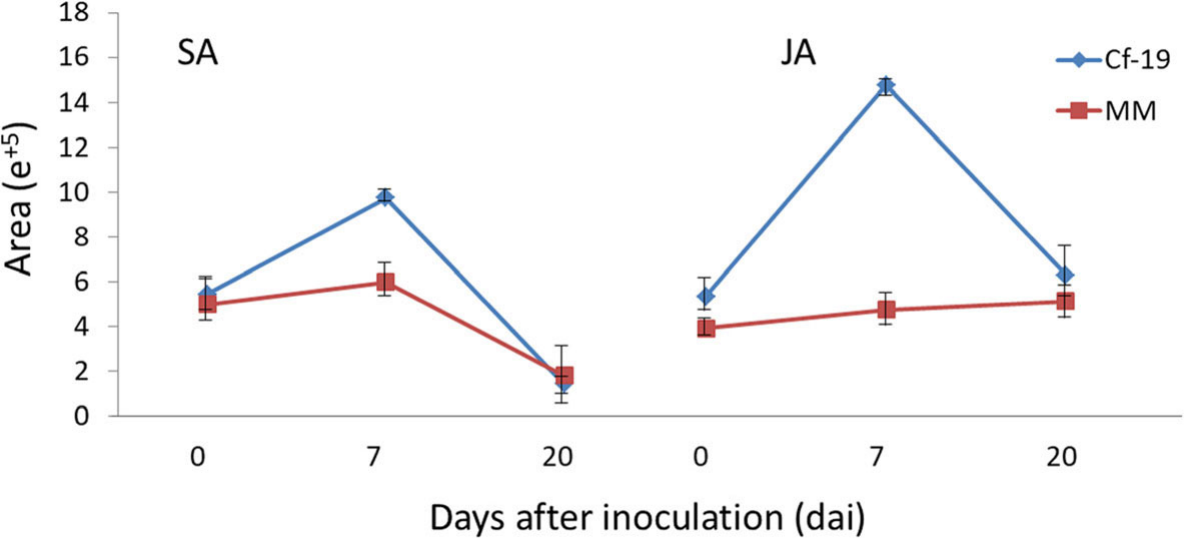
Fluctuations of SA and JA after *C. fulvum* infection in CGN18423 plants and MM plants. SA: salicylic acid, JA: jasmonic acid, Cf-19: CGN18423, MM: Moneymaker

The results of “key regulatory points” identification in the “plant hormone signal transduction pathway” and gene expression pattern analyses indicate that “JAZ” is an important factor involved in the resistance response process. In addition, changes in “JAZ” may affect JA and salicylic acid (SA) functions and contents, and JA and SA are very important hormones involved in plant immunity. To further explore the relevant phenomenon behind changes in “JAZ”, we measured SA and JA contents. The results showed that the SA and JA contents fluctuated differently in the different plants in response to inoculation (Fig. 7). The SA contents increased and peaked at 7 dai, after which the contents deceased and reached minimum values in both CGN18423 and MM plants at 20 dai. Although the change patterns are similar between the resistant and susceptible plants, there was more SA in the resistant plants than in the susceptible plants. The JA content differed dramatically between the resistant and susceptible plants; the JA content increased to a very high level at 7 dai but then decreased to nearly the initial level at 20 dai in CGN18423, while it continued to increase slightly during the whole process from 0 to 20 dai in MM.

## Discussion

### Gene expression changed most actively at the early stage of plant-pathogen interaction

Throughout the whole process of plant-pathogen interactions, the seventh day after *C. fulvum* inoculation is an important time point; this point separates the whole interaction process into two stages, the early stage and the late stage, according to the appearance of necrotic lesions, which were qualified as hypersensitive-like symptoms, and according to the results of gene expression changes [28]. On the basis of the different stages and different materials, two kinds of comparisons, vertical comparisons (gene expression comparisons between resistant plants and susceptible plants at the same time points) and horizontal comparisons (gene expression comparisons within the same plants between different time points), were carried out. The results showed that, in the vertical comparisons, there were more DEGs in the susceptible plants than in the resistant plants at the early stage, while fewer DEGs were detected in the susceptible plants than in the resistant plants at the late stage. Gene expression changes were relatively small in the resistant plants compared to the susceptible plants. In the horizontal comparisons, the gene expression patterns were very different between the early stage and late stage in both resistant and susceptible plants. In general, the expression levels of many genes changed at the early stage, although the expression levels of some of these genes recovered to their initial levels at the late stage. These comparison results indicated that, compared with the susceptible plants, the performance of the resistant plants was relatively stable and that the early stage represents very important response processes for both compatible interactions and incompatible interactions. Similar results were also reported for other Cf gene-mediated resistance responses [13, 14] and other plant-pathogen interactions [29, 30].

### Cf*-19*-mediated resistance was regulated by both SA and JA

We measured SA and JA contents at 0, 7 and 20 dai and found that the JA content increased to very high levels at 7 dai in the resistant plants. This interesting phenomenon was also reported for the Cf*-10*-mediated resistance response [14]. Furthermore, the expression levels of genes at the “JAZ” protein point were upregulated significantly in the resistant plants at 7 and 20 dai. A “JAZ” gene, Solyc12g009220.1, whose expression was upregulated at 7 and 20 dai in this study, was also found to be upregulated in Cf*-12* tomato plants after *C. fulvum* infection [13]. “JAZ” is one of the four key regulatory points we screened in this study. JAZs are direct targets of the SCF^COI1^ E3 ubiquitin ligase and negatively regulate the key transcriptional activator of jasmonate responses, MYC2 [31]. JAZ protein expression suppresses JA activation and indirectly promotes SA activation. Our results indicated that both the JA and SA hormone signalling pathways are involved in the Cf*-19*-mediated resistance response and that “JAZ” plays a very important role in regulating JA and SA during the whole defence process.

### Other important hormone regulation involvement in Cf*-19*-mediated resistance

Plants produce a wide variety of hormones, including auxins (Auxins), gibberellins (GAs), abscisic acid (ABA), cytokinins (CKs), SA, ethylene (ET), JA, brassinosteroids (BRs) and peptide hormones. The infection of plants by various pathogens results in changes in the levels of various phytohormones [32, 33]. In the present study, the “plant hormone signal transduction” pathway, identified from pathways with a *P*-value <0.05, was unique to CGN18423 in the 0–7 dai comparison. DEGs were detected in pathways of eight different hormones, including Auxin, CK, GA, ABA, ET, BR, JA and SA, which revealed a complex signal transduction network. Comparison analysis revealed four key regulatory points for the Auxin, CK, BR and JA hormone signalling pathways based on their resistance response-related change patterns. In addition to the above-mentioned “JAZ”, three other key regulatory points in the “plant hormone signal transduction” pathway were identified: “SAUR” in the “tryptophan metabolism” pathway, “A-ARR” in the “zeatin biosynthesis” pathway and “BR-INSENSITIVE 1 (BRI-1)” in the “brassinosteroid biosynthesis” pathway. All these key points were involved in plant cell morphological changes, such as cell enlargement and cell division. These results suggest that cell morphological changes are also related to the defence process.

### Early Ca^2+^ signalling processes are different between compatible interactions and incompatible interactions

Calmodulin (CaM) and calmodulin-like (CML) proteins are two kinds of calcium (Ca^2+^)-sensing proteins that are involved in Ca^2+^ signalling processes. These proteins play important roles in plant immunity, especially during the early stage of the resistant response [34]. In this study, the most marked difference in the expression patterns of CaM/CML genes appeared in the 0–7 dai comparison between resistant plants and susceptible plants. The expression levels of many genes were upregulated significantly in the susceptible plants but not in the resistant plants, suggesting that calcium signal transduction during the initiation of pathogen infection differs between CGN18423 and MM plants. Previous studies reported PAMP-induced calcium spiking differs from that induced during symbiosis [35, 36]. Our result may indicate that effector-induced calcium spiking also differs from that induced during symbiosis.

### PTI is involved in the resistance response of tomato carrying Cf*-19* and *C. fulvum* interaction

There are two levels of defence responses triggered by microbial recognition in plants. The first level is PAMP, which leads to PTI [37]. The second level involves intracellular immune receptors that recognize pathogen virulence effectors secreted within host cells, thereby inducing ETI [4]. The Cf/Avr interaction is a model for ETI studies, while the expression levels of many BRI1 DEGs and CERK1 DEGs, which are important components of PTI [38–40], were upregulated significantly in the 0–7 dai comparisons in the present study. The expression patterns of these DEGs are consistent with those during the PTI process [39, 41]. Members of the complex family of WRKY TFs have been implicated in the regulation of transcriptional reprogramming associated with plant immune responses. In this study, the expression of many WRKY TFs was found to be upregulated very significantly during the early stage of interaction. Some of these WRKY TFs play important roles in the PTI process [42–45]. The mentioning of all of the PTI-related genes above indicates that PTI is involved in the early response in plants that carry Cf*-19* and that are infected with *C. fulvum*.

### A presumed model of the Cf*-19*-dependent resistance response

**Fig. 8:**
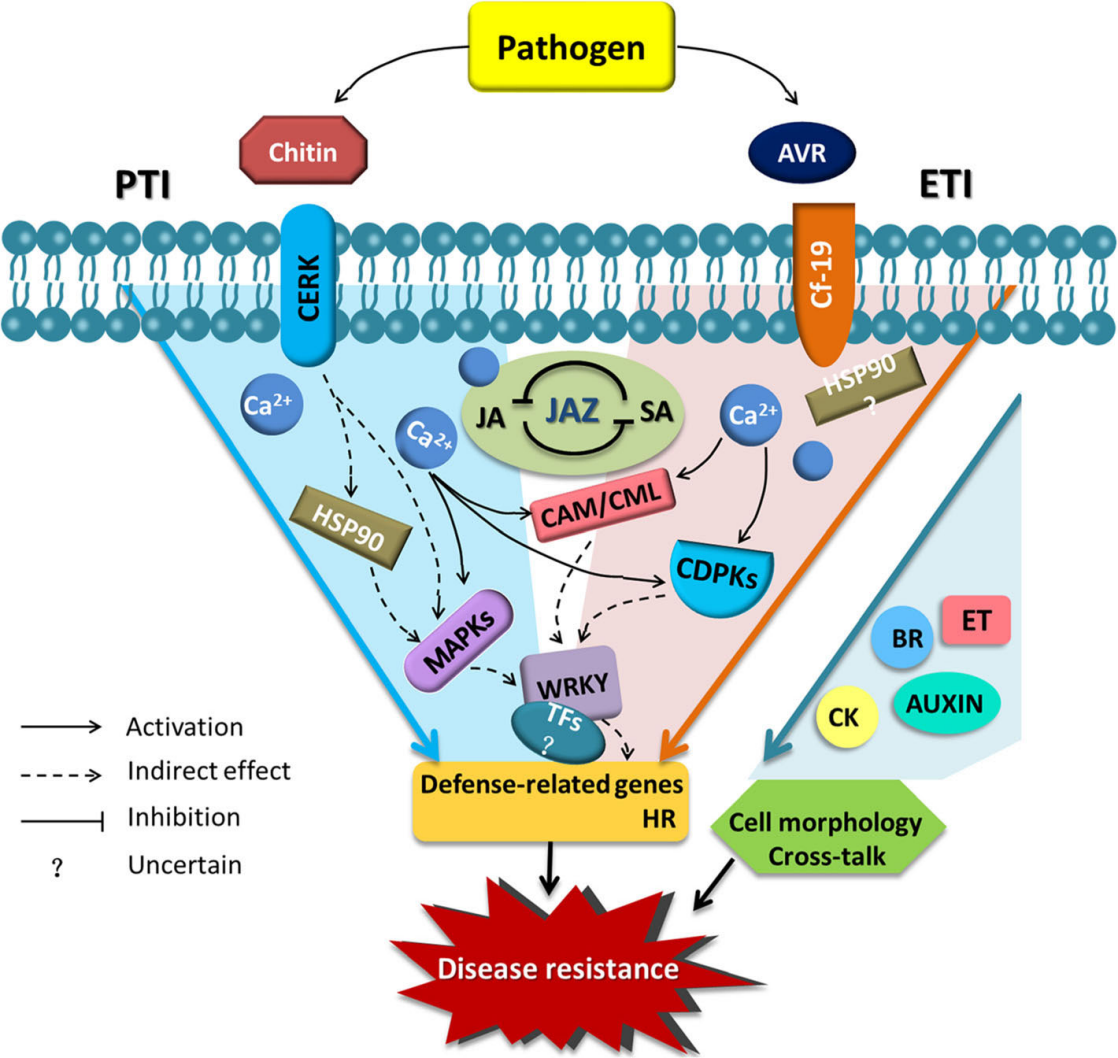
Model of the resistance response of tomato plants that carry Cf*-19* and that are infected with *C. fulvum*. Both PTI and ETI are involved in the resistance response. The balance between JA and SA, which is maintained by JAZ, is a main regulatory factor in the response process. PAMP: pathogen-associated molecular pattern; PTI: PAMP-triggered immunity; ETI: effector-triggered immunity; CAM: calmodulin; CML: calmodulin-like; MAPK: mitogen-activated protein kinases; CDPK: calcium-dependent protein kinase; HSP90: Heat shock protein 90; JA: jasmonic acid; SA: salicylic acid; JAZ: jasmonate ZIM-domain; GA: gibberellin; ABA: abscisic acid; CK: cytokinin; ET: ethylene; BR: brassinosteroid; HR: hypersensitive response

On the basis of the findings in the present study and in the literature on plant immunity [46, 47], we speculated about the general process of the Cf*-19-*dependent resistance response. As shown in Fig. 8, when plants carrying Cf*-19* were infected by the *C. fulvum* pathogen, both ETI and PTI were triggered. ETI was triggered by the recognition between AVR and the Cf-19 protein, and PTI was triggered by the recognition between CERK and chitin. The recognition was followed by an influx of Ca^2+^, some of which bound to different CAM/CML proteins to active downstream signalling pathways. The increase in cytosolic Ca^2+^ concentration could also create the proper conditions for the activation of CDPK signalling components. Mitogen-activated protein kinase (MAPK) modules could also be activated following upstream changes, including activation of heat shock protein 90 (HSP90). Many TFs, especially WRKYs, accumulated and indirectly regulated the expression of defence-related genes. These changes ultimately resulted in the expression of defence-related genes and the HR. During the whole resistance response process, both SA and JA play important regulatory roles, and JAZ is a key factor in maintaining the balance between these two hormones. Additionally, CKs, BRs, Auxin and ET play important roles in the resistance response by altering cell morphology or by affecting other cross-talk involved in the hormone regulatory network.

## Conclusions

In this study, a total of 35,136 novel transcripts were obtained after transcriptome reconstruction. DEG analysis revealed that the initial responses are very different between the resistant and susceptible plants and are very important for plant immunity. Moreover, early Ca^2+^ signalling processes are different between compatible interactions and incompatible interactions. The “plant hormone signal transduction” pathway and the “plant pathogen interaction” pathway, which contain ten key regulatory points, are important for the Cf*-19*-mediated resistance response, especially at the initial stage, and both PTI and ETI are involved in the resistance response. Last, the balance between JA and SA maintained by JAZ proteins may play a key role in regulating the Cf*-19*-dependent resistance response.

## Methods

### Plant material treatments

The resistant line CGN18423 (carrying the Cf*-19* gene, provided by the Institute of Vegetable and Flowers, Chinese Academy of Agricultural Sciences) and the susceptible line MM (carrying the Cf*-0* gene) were grown at Horticultural Experimental Station of Northeast Agricultural University. Thirty CGN18423 seedlings and 30 MM seedlings at the five-to-six-leaf stage were inoculated with *C. fulvum* race 1.2.3.4. Leaf samples were collected before inoculation and at 7 and 20 dai. All the leaf samples were immediately frozen in liquid nitrogen and stored at − 80 °C prior to RNA extraction. Three biological replicates per treatment were prepared.

### RNA extraction and Illumina sequencing

Total RNA was extracted from the leaf samples using a plant RNA mini kit (Watson, China) according to the manufacturer’s instructions. The extracted total RNA was given to BGI Tech (Shenzhen, China) company for high-throughput sequencing. The following detailed experimental methods were provided by the company. The mRNA samples were isolated from the total RNA samples (treated with DNase I) by using oligo (dT). Eighteen cDNA libraries were constructed using purified mRNAs. The libraries were then sequenced using an Illumina HiSeq4000 machine according to the Illumina protocols [13, 48].

### Mapping of RNA-seq reads and DEG identification

The reads were filtered by SOAPnuke software. Reads with adapters or with a sequencing quality less than 5 were removed [13, 48]. The filtered reads were mapped to the *S. lycopersicum* genome (Tomato Genome Consortium, 2012). Gene expression levels were quantified by normalizing read counts to the aligned fragments per kilobase of transcript per million mapped reads (FPKM) [49, 50].

Hierarchical indexing for spliced alignment of transcripts (HISAT) was used to align paired-end clean reads to the reference genome [13, 51]. The NOIseq methods in conjunction with a noisy distribution model were used to detect DEGs [52, 53]. The false discovery rate (FDR) with a corrected *P* value ≤0.05 and an absolute value of log2Ratio ≥ 1 were used as the thresholds to define significant DEGs [54]. Novel transcripts were reconstructed using StringTie [55].

### GO enrichment and KEGG analyses of DEGs

Significantly enriched GO terms (*P*-value <0.05) were identified by the GOseq R package analyses [56]. Significantly enriched KEGG metabolic pathways were detected by using a hypergeometric test of KOBAS (v2.0) (http://kobas.cbi.pku.edu.cn/) analyses [57].

### qRT-PCR analysis

Fifteen DEGs were selected for expression verification. The primers (Additional file 2: Table S2) were designed with Primer 5.0 software. qRT-PCR (including the reaction mixture and thermal conditions) was performed in accordance with the methods of our previous study [27]. The reactions were carried out on an iQ5 system (Bio-Rad, USA). The data were analysed via the 2^–∆∆CT^ method [58], with *EFα1* gene used for normalization [59].

### Hormone measurements

Endogenous SA and JA were extracted from leaves using the modified methods of Llugany et al. (2013) [60] and Liu et al. (2018) [14]. The SA and JA concentrations were subsequently measured using liquid chromatography-mass spectrometry (LC-MS) according to the manufacturer’s instructions (AB Sciex QTRAP 5500, USA).

## Supplementary information

**Additional file 1: Table S1.** Summary of sequencing reads after filtering. Detailed information, including the Illumina Q20 and Q30 quality scores of the transcripts, are shown in Table S1.

**Additional file 2: Table S2.** Primers used for qRT-PCR analysis. The sequences of primers used for qRT-PCR are shown in Table S2.

**Additional file 3: Figure S1.** GO classification of DEGs. The X axis represents the number of DEGs. The Y axis represents the GO terms. 0–7 dai: comparison between 0 and 7 dai; 7–20 dai: comparison between 7 and 20 dai; 0–20 dai: comparison between 0 and 20 dai; dai: days after inoculation.

**Additional file 4: Figure S2.** Key regulatory points in the plant hormone signal transduction pathway. Pathway regulatory points containing genes that exhibit upregulated expression patterns in Cf-19 in the 0–7 dai and 0–20 dai comparisons (vertical comparisons, shown as four rectangles) or that exhibit upregulated expression patterns in CGN18423 plants compared with MM plants at the same interaction stages (lateral comparisons, shown as three rounds) were identified as important regulatory points. The yellow circle represents the vertical comparison result, and the blue circles represent the lateral comparison results.

**Additional file 5: Figure S3.** Key regulatory points in the plant pathogen interaction pathway. Pathway regulatory points containing genes that exhibit upregulated expression patterns in Cf-19 in the 0–7 dai and 0–20 dai comparisons (vertical comparisons, shown as four rectangles) or that exhibit upregulated expression patterns in CGN18423 plants compared with MM plants at the same interaction stages (lateral comparisons, shown as three rounds) were identified as important regulatory points. The yellow circle represents the vertical comparison result, and the blue circles represent the lateral comparison results.

## Data Availability

The clean data of all samples have been submitted to GenBank of the National Center for Biotechnology Information (NCBI, https://www.ncbi.nlm.nih.gov), and the Sequence Read Archive (SRA) accession number is SRP157120.

## Abbreviations

ABA: Abscisic Acid
*Avr*: Avirulence gene
BRI-1: BR-INSENSITIVE 1
BRs: Brassinosteroids
*C. fulvum*: *Cladosporium fulvum*
CaM: Calmodulin
cDNA-AFLP: cDNA-Amplified Fragment Length Polymorphism
CDPK: Calcium-Dependent Protein Kinase
CML: Calmodulin-Like
dai: days after inoculation
DEGs: Differentially Expressed Genes
ET: Ethylene
ETI: Effector-Triggered Immunity
Gas: Gibberellins
GO: Gene Ontology
*Hcr2*: Homologues of *Cladosporium* resistance gene Cf*-2*
*Hcr9*: Homologues of *Cladosporium* resistance gene Cf*-9*
HR: Hypersensitive Response
JA: Jasmonic Acid
JAZ: Jasmonate ZIM-domain
KEGG: Kyoto Encyclopedia of Genes and Genomes
LRR: Leucine-Rich Repeat
PAMP: Pathogen-Associated Molecular Pattern
PRRs: Pattern Recognition Receptors
PTI: PAMP-Triggered Immunity
qRT-PCR: Quantitative real-time PCR
R gene: Resistance gene
RLPs: Receptor-Like Proteins
SA: Salicylic Acid
TDFs: Transcript-Derived Fragments
TF: Transcription Factor
MAPK: Mitogen-Activated Protein Kinase
HSP90: Heat Shock Protein 90
QC: Quality Control
RSEM: RNA-Seq by Expectation Maximization
FPKM: Fragments Per Kilobase Per Million Mapped Reads
HISAT: Hierarchical Indexing for Spliced Alignment of Transcripts
LC-MS: Liquid Chromatography-Mass Spectrometry
NCBI: National Center for Biotechnology Information
SGN: SOL Genomics Network
