# Supplementary material for: Transcriptome profiling reveals the response process of tomato carrying Cf-19 and Cladosporium fulvum interaction
Source: BMC Plant Biol. 2019 Dec 19;19:572. doi: 10.1186/s12870-019-2150-y (PMC6923989; doi:10.1186/s12870-019-2150-y)
Supplement: Supplementary file 1 — Additional file 1: Table S1. Summary of sequencing reads after filtering. Detailed information, including the Illumina Q20 and Q30 quality scores of the transcripts, are shown in Table S1. [file 12870_2019_2150_MOESM1_ESM.doc]

Table S1 Summary of sequencing reads after filtering

| Sample | Total Raw  Read s (Mb) | Total Clean  Read s (Mb) | Total Clean  Base s (Gb) | Clean Reads  Q20 (%) | Clean Reads  Q30 (%) | Clean Reads  Ratio (%) |
| --- | --- | --- | --- | --- | --- | --- |
| CF19 Day 0 1 | 39.19 | 29.75 | 4.46 | 97.54 | 93.09 | 75.92 |
| CF19 Day 0 2 | 37.56 | 29.93 | 4.49 | 97.58 | 93.14 | 79.70 |
| CF19 Day 0 3 | 38.73 | 28.82 | 4.32 | 97.34 | 92.61 | 74.40 |
| CF19 Day 20 1 | 39.19 | 30.16 | 4.52 | 97.54 | 93.11 | 76.97 |
| CF19 Day 20 2 | 37.56 | 29.81 | 4.47 | 97.58 | 93.16 | 79.39 |
| CF19 Day 20 3 | 42.46 | 30.45 | 4.57 | 97.39 | 92.71 | 71.72 |
| CF19 Day7 1 | 39.19 | 30.15 | 4.52 | 97.41 | 92.78 | 76.93 |
| CF19 Day7 2 | 33.85 | 28.78 | 4.32 | 95.69 | 89.67 | 85.03 |
| CF19 Day7 3 | 37.33 | 27.46 | 4.12 | 97.36 | 92.66 | 73.56 |
| MM Day 0 1 | 40.82 | 29.90 | 4.48 | 97.51 | 93.02 | 73.24 |
| MM Day 0 2 | 38.29 | 28.03 | 4.20 | 97.48 | 92.95 | 73.21 |
| MM Day 0 3 | 40.82 | 30.17 | 4.53 | 97.70 | 93.43 | 73.90 |
| MM Day20 1 | 39.19 | 29.76 | 4.46 | 97.44 | 92.83 | 75.93 |
| MM Day20 2 | 36.10 | 27.22 | 4.08 | 97.41 | 92.75 | 75.39 |
| MM Day20 3 | 33.14 | 27.94 | 4.19 | 95.62 | 89.51 | 84.31 |
| MM Day7 1 | 40.82 | 30.28 | 4.54 | 97.31 | 92.56 | 74.18 |
| MM Day7 2 | 39.19 | 29.69 | 4.45 | 97.62 | 93.25 | 75.75 |
| MM Day7 3 | 39.19 | 29.37 | 4.41 | 97.61 | 93.22 | 74.94 |
