# Supplementary material for: Transcriptome profiling reveals the response process of tomato carrying Cf-19 and Cladosporium fulvum interaction
Source: BMC Plant Biol. 2019 Dec 19;19:572. doi: 10.1186/s12870-019-2150-y (PMC6923989; doi:10.1186/s12870-019-2150-y)
Supplement: Supplementary file 2 — Additional file 2: Table S2. Primers used for qRT-PCR analysis. The sequences of primers used for qRT-PCR are shown in Table S2. [file 12870_2019_2150_MOESM2_ESM.docx]

**Table S2.** Primers used for qRT-PCR analysis

| Primer Name | | Forward primer sequence (5′–3′) | Reverse primer sequence (5′–3′) |
| --- | --- | --- | --- |
| Solyc02g071220.3.1 | | GCCGGAAATGACTGGTTATGA | CTGGTTCTGTCAGGTGAAATGC |
| Solyc11g018775.1.1 | | CTCTCTTGCTTGATGAAACTCCA | AGCACACGATACAACTCCAGG |
| Solyc11g017270.2.1 | | TTTGGGGTTGTTCTGCTGGA | TCATTTTTGGCCGGCCTTCT |
| Solyc07g055380.2.1 | | GGACTCCCCCTAGCATTGAA | CGCTCCACTTCACTTAACCA |
| Solyc10g084380.1.1 | | AGCGCAACTTGTCAGACGTA | GGCATCAGTGTGCATTCCTG |
| Solyc02g089610.2.1 | | TTCCCACATCGCCACTTCTC | TATTGGCATCGGCAGGTTCA |
| Solyc04g007500.1.1 | | TTCAGGTTTAGCCGGAGTCG | ACCGGCAAATCGGACAGTTA |
| Solyc01g005160.3.1 | TGCTTCAACAGAGGAAGTGATCT | ACAAGCCCTTCTAATGCACCT | |
| Solyc10g052880.1.1 | GGGCCGATACCAAGTTCGAT | GTGCCCTACCTCAAGGAACC | |
| Solyc12g056410.2.1 | TCGTGGTTTTAGGGCTGCTT | TCGTCAGTTTCGATGCGACT | |
| Solyc03g113720.3.1 | TCGGTGTTGAGCAAGATCCC | CCTCGCCCCTCAGAATGAAG | |
| Solyc05g006420.3.1 | CTGTGGATAGTGGGATGAGAGC | CTGCCAATTTCACCGGCTTC | |
| Solyc08g036640.3.1 | GGTTTCCGATGCTACTGAGC | TGGCGAAGTTGCTTGAACTC | |
| Solyc12g009220.2.1 | CGTCATCGTCGTCCGTTGAA | TGGGGTTCTGTTTGTTGGCTA | |
| Solyc00g174340.2.1 | GAGTCGGGCCTATGTCTTGG | GAACCACCATCCGTTGTTGC | |
| EFα1 | | CCACCAATCTTGTACACATCC | AGACCACCAAGTACTACTGCAC |
