# Supplementary figures and images for: Transcriptome profiling reveals the response process of tomato carrying Cf-19 and Cladosporium fulvum interaction
Source: BMC Plant Biol. 2019 Dec 19;19:572. doi: 10.1186/s12870-019-2150-y (PMC6923989; doi:10.1186/s12870-019-2150-y)

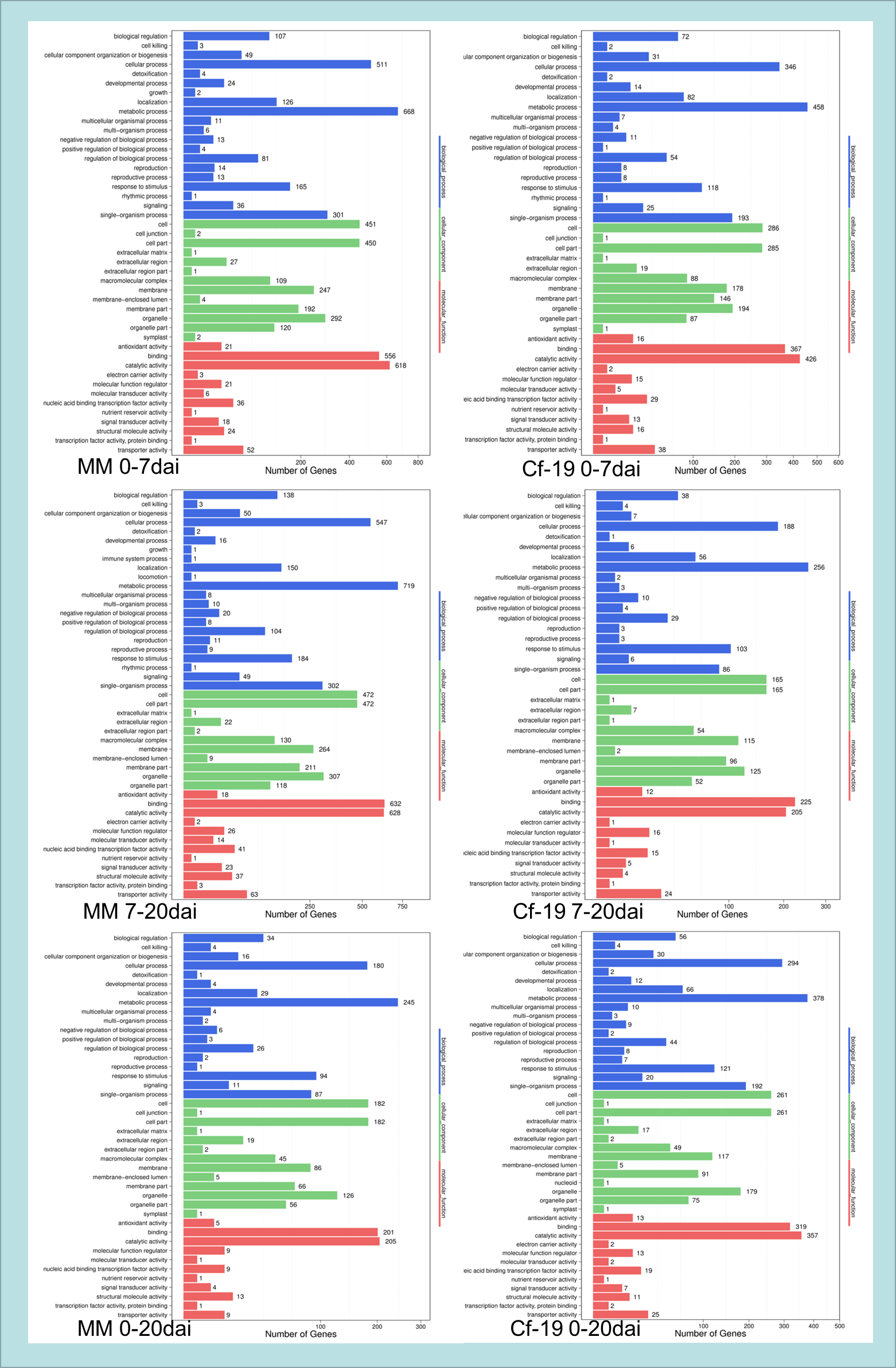

Supplement: Supplementary file 3 — Additional file 3: Figure S1. GO classification of DEGs. The X axis represents the number of DEGs. The Y axis represents the GO terms. 0–7 dai: comparison between 0 and 7 dai; 7–20 dai: comparison between 7 and 20 dai; 0–20 dai: comparison between 0 and 20 dai; dai: days after inoculation. [file 12870_2019_2150_MOESM3_ESM.tif]

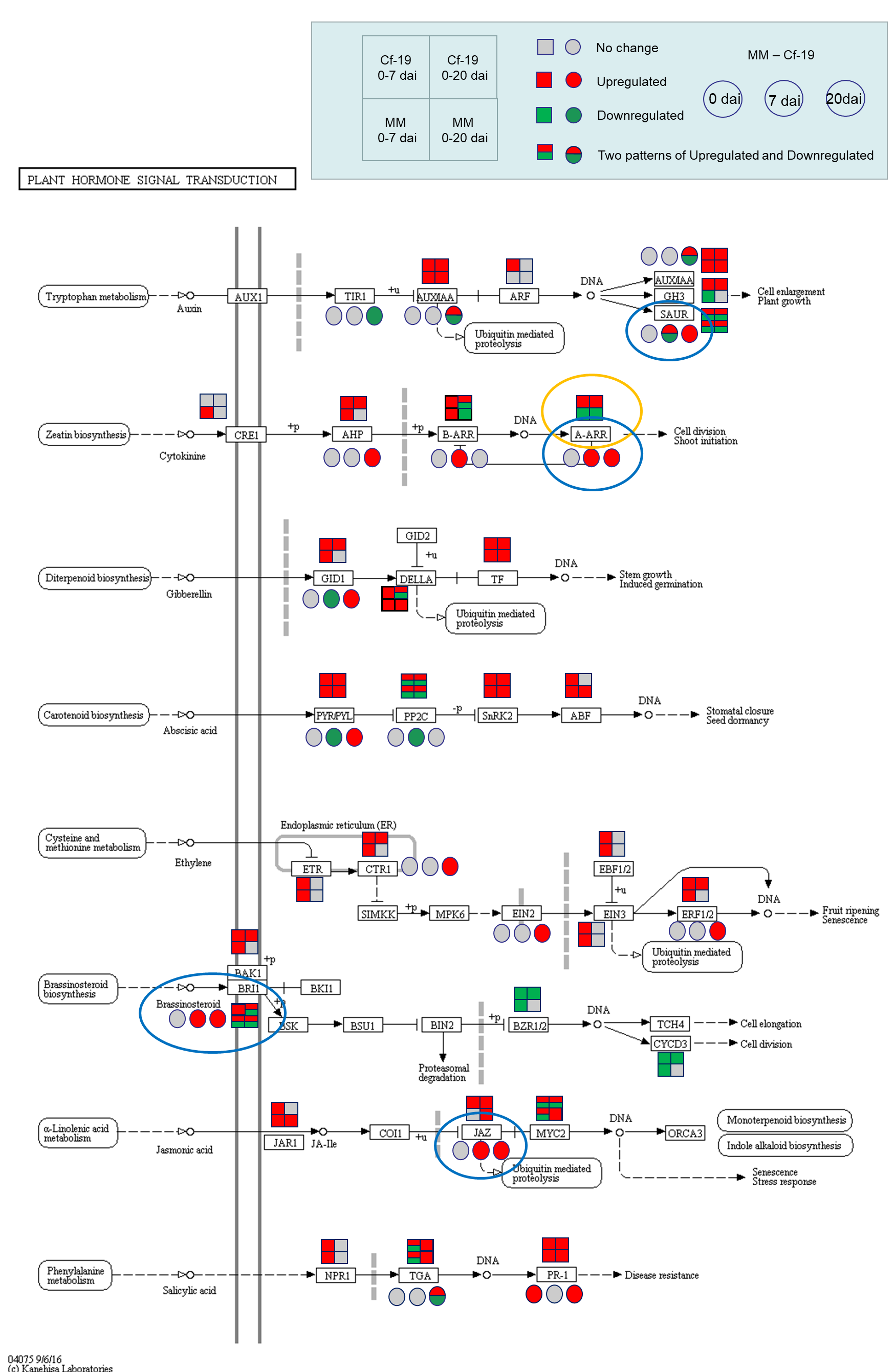

Supplement: Supplementary file 4 — Additional file 4: Figure S2. Key regulatory points in the plant hormone signal transduction pathway. Pathway regulatory points containing genes that exhibit upregulated expression patterns in Cf-19 in the 0–7 dai and 0–20 dai comparisons (vertical comparisons, shown as four rectangles) or that exhibit upregulated expression patterns in CGN18423 plants compared with MM plants at the same interaction stages (lateral comparisons, shown as three rounds) were identified as important regulatory points. The yellow circle represents the vertical comparison result, and the blue circles represent the lateral comparison results. [file 12870_2019_2150_MOESM4_ESM.tif]

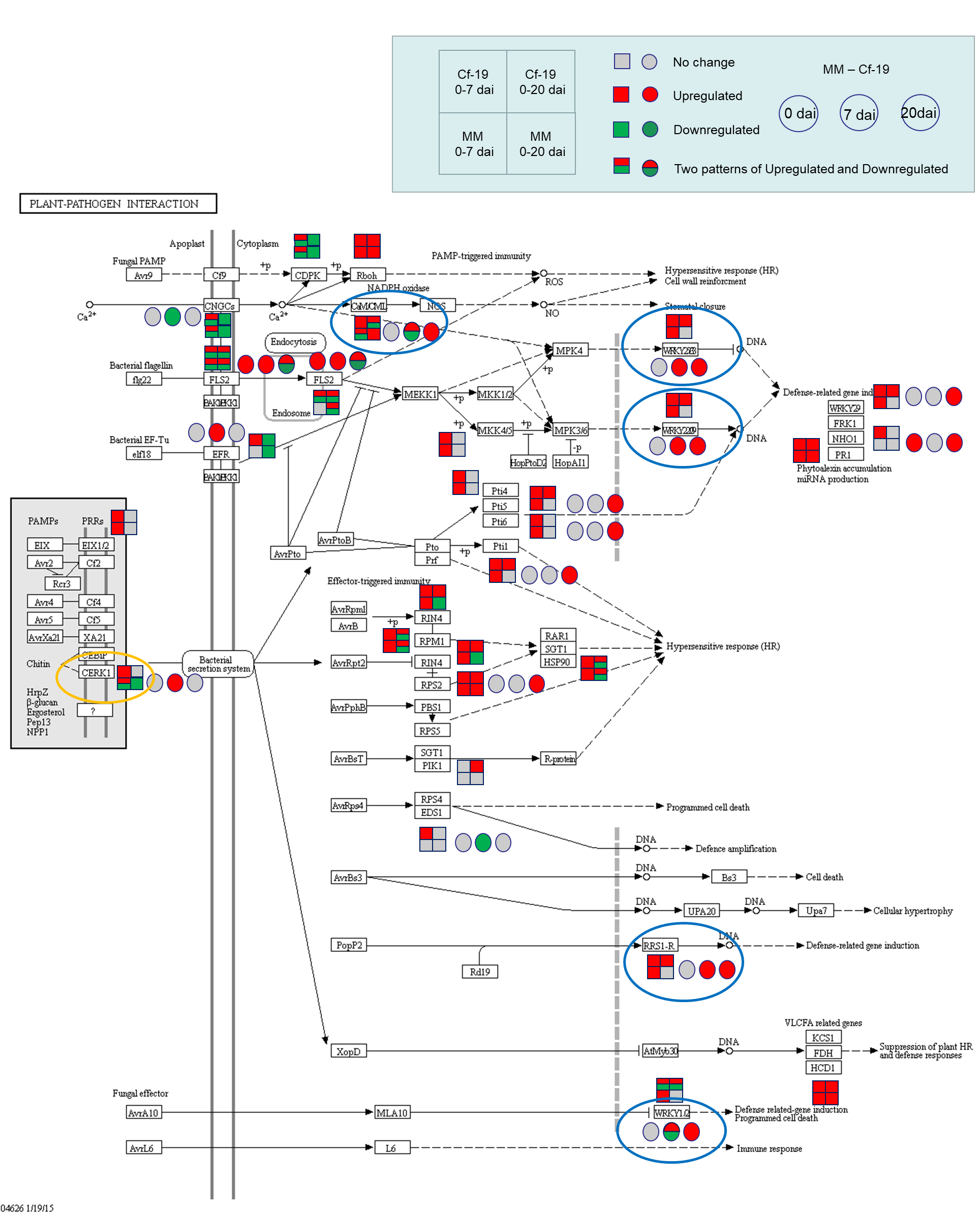

Supplement: Supplementary file 5 — Additional file 5: Figure S3. Key regulatory points in the plant pathogen interaction pathway. Pathway regulatory points containing genes that exhibit upregulated expression patterns in Cf-19 in the 0–7 dai and 0–20 dai comparisons (vertical comparisons, shown as four rectangles) or that exhibit upregulated expression patterns in CGN18423 plants compared with MM plants at the same interaction stages (lateral comparisons, shown as three rounds) were identified as important regulatory points. The yellow circle represents the vertical comparison result, and the blue circles represent the lateral comparison results. [file 12870_2019_2150_MOESM5_ESM.tif]
